# GSK-3β downregulates Nrf2 in cultured cortical neurons and in a rat model of cerebral ischemia-reperfusion

**DOI:** 10.1038/srep20196

**Published:** 2016-02-03

**Authors:** Xi Chen, Yuanling Liu, Jin Zhu, Shipeng Lei, Yuan Dong, Lingyu Li, Beibei Jiang, Li Tan, Jingxian Wu, Shanshan Yu, Yong Zhao

**Affiliations:** 1 Department of Pathology, Chongqing Medical University, 400016 Chongqing, People’s Republic of China; 2 Institute of Neuroscience, Chongqing Medical University, 400016 Chongqing, People’s Republic of China; 3 Key Laboratory of Neurobiology, Chongqing Medical University, 400016 Chongqing, People’s Republic of China; 4 Department of Respiratory Medicine, Jiangjin Center Hospital, 402260, Chongqing, People’s Republic of China; 5 Department of Forensic Medicine, Chongqing Medical University, 400016 Chongqing, People’s Republic of China

## Abstract

The NF-E2-related factor 2 (Nrf2)/antioxidant response element (ARE) pathway plays a critical role in protecting against oxidative stress in brain ischemia and reperfusion injury. Glycogen synthase kinase 3β (GSK-3β) may play a critical role in regulating Nrf2 in a Kelch-like ECH-associated protein 1 (Keap1)-independent manner. However, the relationship between GSK-3β and Nrf2 in brain ischemia and reperfusion injury is not clear. In this study, we explored the mechanisms through which GSK-3β regulates Nrf2 and Nrf-2/ARE pathways *in vitro* and *in vivo*. We used oxygen and glucose deprivation/reoxygenation (OGD/R) in primary cultured cortical neurons and a middle cerebral artery occlusion-reperfusion (MCAO/R) rat model to mimic ischemic insult. In this study, GSK-3β siRNA and inhibitors (SB216763 and LiCl) were used to inhibit GSK-3β *in vitro* and *in vivo*. After inhibiting GSK-3β, expression of total and nuclear Nrf2, Nrf2-ARE binding activity, and expression of Nrf2/ARE pathway-driven genes HO-1 and NQO-1 increased. Overexpression of GSK-3β yielded opposite results. These results suggest that GSK-3β downregulates Nrf2 and the Nrf2/ARE pathway in brain ischemia and reperfusion injury. GSK-3β may be an endogenous antioxidant relevant protein, and may represent a new therapeutic target in treatment of ischemia and reperfusion injury.

In brain ischemic and reperfusion injury, oxidative stress is caused by an excess of intracellular oxidants or reactive oxygen species (ROS). Endogenous antioxidant proteins can eliminate intracellular oxidants and ROS. Endogenous antioxidant proteins play a crucial role in antioxidant stress^1^. Therefore, improving expression of endogenous antioxidant proteins may be an effective way to reduce cell and brain tissue damage. Recent evidence indicates that endogenous antioxidant systems have a close relationship with Nuclear factor (erythroid-derived 2)-like 2 (Nrf2)^2^.

Nrf2 is a Cap’n’Collar (CNC) basic-region leucine zipper (bZIP) transcription factor that plays a crucial role in the endogenous antioxidant stress system through control of antioxidant response element (ARE)-driven genes^3^, which induce expression of some phase II detoxifying enzyme and antioxidant stress genes, such as NAD(P)H: quinone oxidoreductase 1 (NQO1), hemeoxygenase-1(HO-1), glutathione S-transferase (GST), and aldo-keto reductase (AR)^3^,^4^,^5^. In normal cells, Kelch-like ECH-associated protein 1 (Keap1) regulates the level of Nrf2 protein by Keap1/Cullin 3/Rbx1 (Ring-box 1 protein) ubiquitination and degradation^6^,^7^,^8^. Recent evidence indicates that GSK-3β plays a critical role in regulating and degrading Nrf2 in a Keap1-independent manner^9^,^10^.

GSK-3β is a serine/threonine protein kinase, which is known to be involved in a number of human disorders such as diabetes, cancer, oxidative stress, psychiatric disorders, and Alzheimer’s disease^11^. GSK-3β participates in neuronal death through PI3K/AKT and Wnt/β-catenin signaling pathways^12^. GSK-3β is highly active in resting cells and usually inhibits downstream pathways^11^. Activation of GSK-3β is correlated with phosphorylation at tyrosine 216. Regulation of GSK-3β usually depends on phosphorylation within the amino-terminal domain of GSK-3β (Ser 9) and results in inactivation of GSK-3β by several kinases including Akt, PKA, and PKC^11^. Recent studies have shown that inhibiting GSK-3β can reduce Nrf2 by nuclear export and degradation of Nrf2 in liver cancer cells and improve the rate of cell survival during the late phase of oxidative stress^9^,^10^. However, there have been no studies showing how GSK-3β regulates Nrf2 in brain ischemia and reperfusion injury.

In this study, oxygen–glucose deprivation followed by recovery (OGD/R) and middle cerebral artery occlusion-reperfusion (MCAO/R) were used to mimic ischemic reperfusion insult *in vitro* and *in vivo*, respectively. GSK-3β siRNA and inhibitors (SB216763 and LiCl) were used to inhibit GSK-3β. We found that GSK-3β downregulates expression levels of Nrf2, Nrf2-ARE binding activity, and expression levels of genes downstream of Nrf2/ARE in brain ischemic and reperfusion injury.

## Results

### Expression levels of P-GSK-3β (tyr216), total GSK-3β, β-catenin, and Nrf2 varied with reoxygenation time

**Figure 1:**
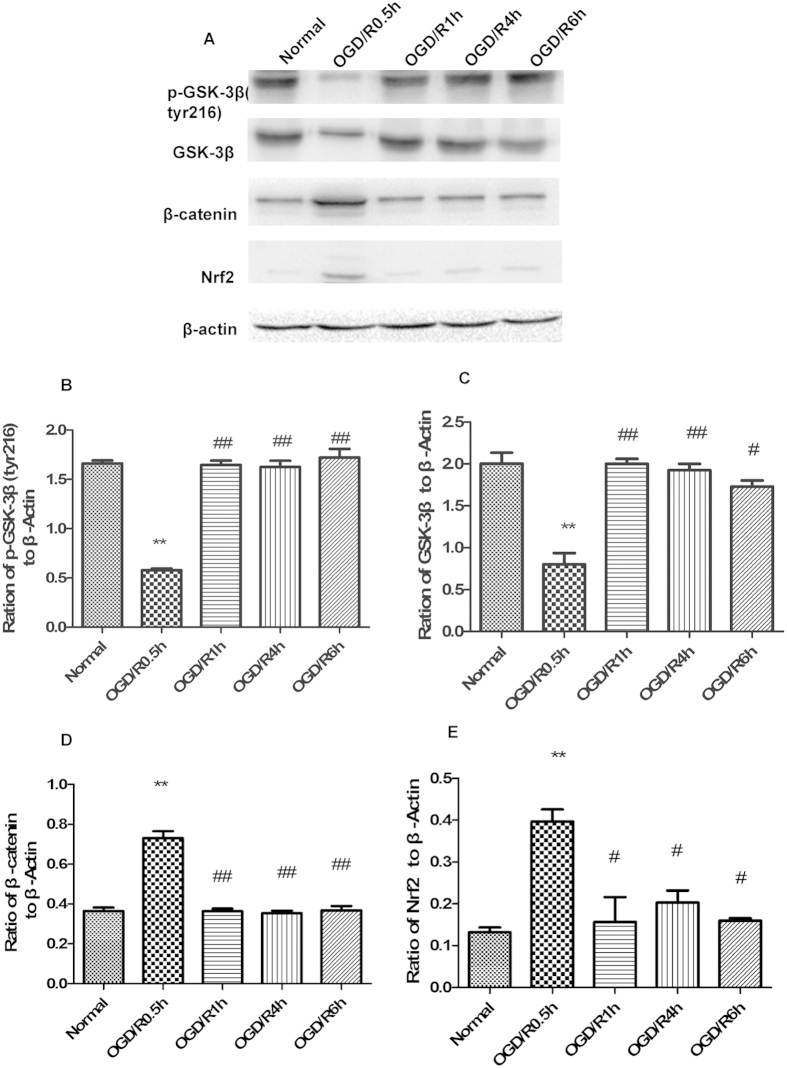
P-GSK-3β (tyr216), total GSK-3β, β-catenin, and Nrf2 expression levels varied with reoxygenation time. After 1.5 h of oxygen and glucose deprivation (OGD), neurons were harvested after 0.5 h, 1 h, 4 h, and 6 h of reoxygenation. (**A**) Western blot analyses of p-GSK-3β (tyr216), total GSK-3β, β-catenin, and Nrf2. (**B–E**) Representative ratios of p-GSK-3β (tyr216), total GSK-3β, β-catenin, and Nrf2 to β-actin. P-GSK-3β (tyr216) underwent a short-term decrease after 0.5 h of reoxygenation and then initially reached the highest level after 1 h of reoxygenation. Total GSK-3β expression levels showed a similar trend. β-catenin and Nrf2 expression levels showed a reverse trend. Bars represent mean ± SEM (n = 4–6). **p < 0.01 vs. Normal, ^#^p < 0.05 vs. 0.5 h reoxygenation, ^##^p < 0.01 vs. 0.5 h reoxygenation.

The activated form of GSK-3β is P-GSK-3β (tyr216). After 1.5 h of OGD, neurons were reoxygenated for 0.5 h, 1 h, 4 h, or 6 h. Western blot analysis was performed to examine expression of P-GSK-3β (tyr216), total GSK-3β, β-catenin, and Nrf2 (Fig. 1). After 0.5 h of reoxygenation, the level of p-GSK-3β (tyr216) was decreased compared with the normal group (0.57 ± 0.016 vs. 1.66 ± 0.03, respectively) (Fig. 1B). The expression level of p-GSK-3β (tyr216) significantly increased to 1.64 ± 0.04 in the 1 h reoxygenation group, and remained at this level in the 4 h and 6 h groups. Total GSK-3β expression showed a similar trend (Fig. 1A). β-catenin levels served as a control for GSK-3β inhibition, and the expression levels yielded opposite results (Fig. 1A). In parallel experiments, we found that Nrf2 expression presented a reverse trend (Fig. 1A). After 0.5 h of reoxygenation, the expression level of Nrf2 was elevated approximately 3-fold. After 1 h, 4 h, and 6 h of reoxygenation, expression of Nrf2 decreased to the normal level. The expression of p-GSK-3β (tyr216) initially reached its highest level after 1 h of reoxygenation. Therefore, to analyze GSK-3β regulation of Nrf2 in brain ischemic and reperfusion injury, we chose 1 h of reoxygenation as the optimum time for this study.

### GSK-3β regulates Nrf2 in cultured neurons after OGD/R

**Figure 2:**
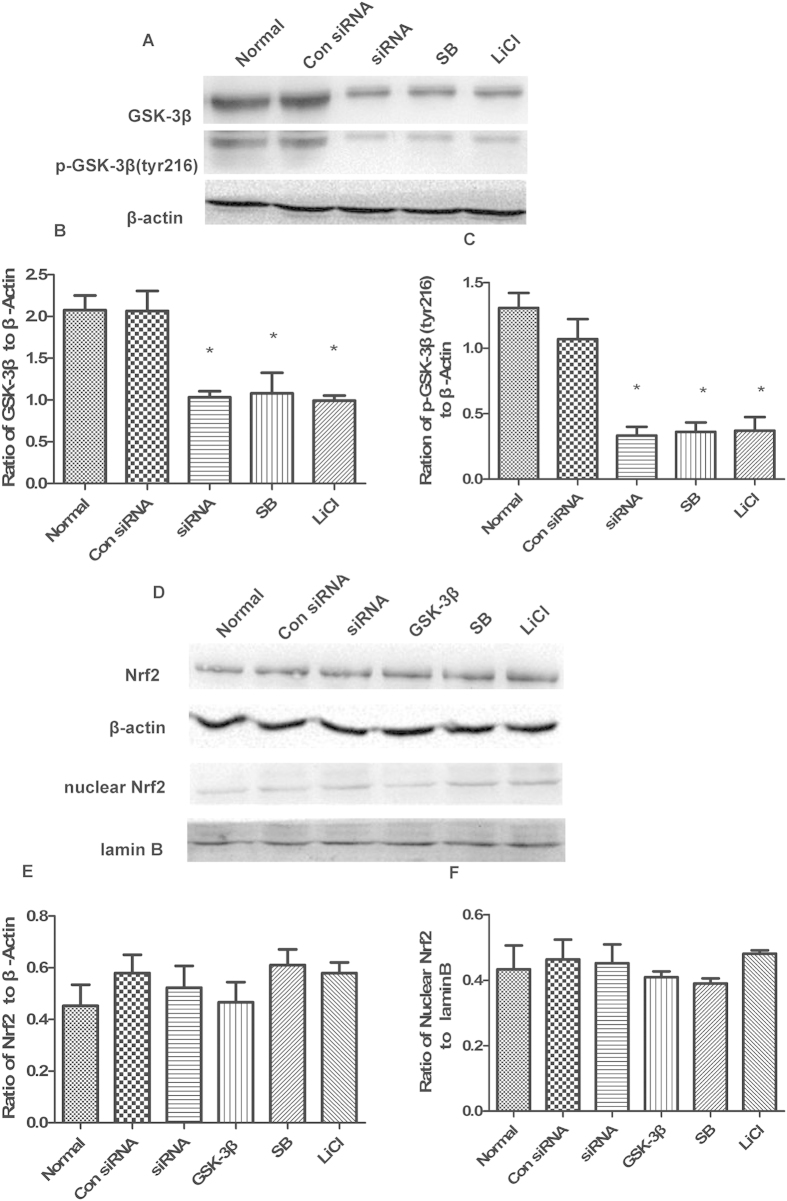
GSK-3 β regulation of Nrf2 in neurons under normal conditions. (**A**) Western blot analysis of GSK-3β and p-GSK-3β (tyr216). (**B,C**) Representative ratios of GSK-3β and p-GSK-3β (tyr216) to β-actin. GSK-3β and p-GSK-3β (tyr216) expression significantly decreased in the siRNA (GSK-3β siRNA) and inhibitor [SB(SB216763) and LiCl] groups compared with the normal group. (**D**) Western blot analysis of Nrf2 and nuclear Nrf2. (**E,F**) Representative ratios of Nrf2 and nuclear Nrf2 to β-actin. There was no significant difference in the expression levels of Nrf2 and nuclear Nrf2 in siRNA, inhibitor, and GSK-3β groups compared with the normal group. Con siRNA indicates control siRNA. Bars represent mean ± SEM (n = 4–6) *p < 0.05 vs. Normal.

**Figure 3:**
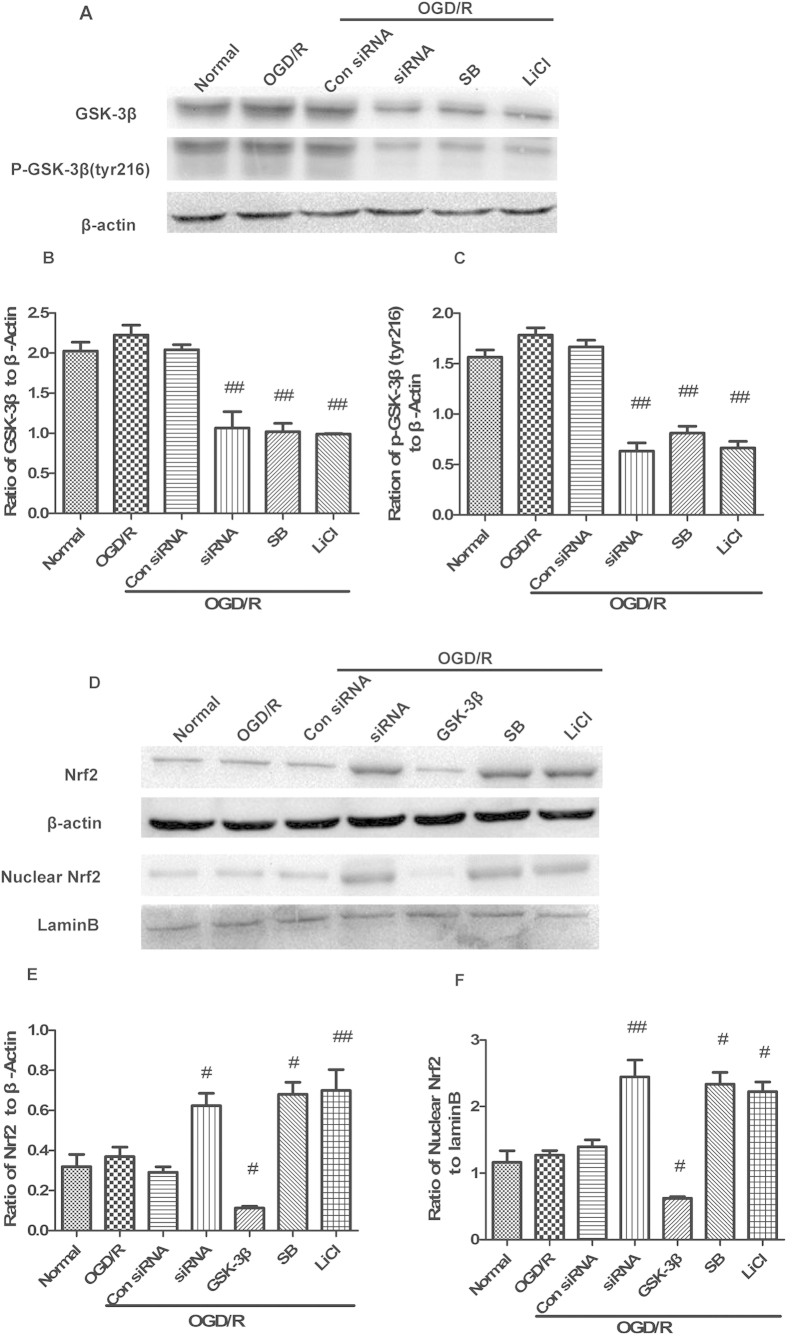
GSK-3β regulates Nrf2 in neurons after oxygen and glucose deprivation/reoxygenation (OGD/R). Cells were subjected to OGD for 1.5 h followed by 1 h of reoxygenation, after which cells were harvested. (**A**) Western blot analyses of GSK-3β and p-GSK-3β (tyr216). (**B,C**) Representative ratios of GSK-3β and p-GSK-3β (tyr216) to β-actin. GSK-3β and p-GSK-3β (tyr216) expression significantly decreased in the siRNA + OGD/R and inhibitor +OGD/R groups compared with the OGD/R group. (**D**) Western blot analysis of Nrf2 and nuclear Nrf2. (**E,F**) Representative ratios of Nrf2 and nuclear Nrf2 to β-actin. Expression levels of Nrf2 and nuclear Nrf2 significantly increased in the siRNA + OGD/R and inhibitor + OGD/R groups. Nrf2 and nuclear Nrf2 expression levels significantly decreased in the GSK-3β + OGD/R group. Bars represent mean ± SEM (n = 4–6). ^#^p < 0.05 vs. OGD/R, ^##^P < 0.01 vs. OGD/R.

**Figure 4:**
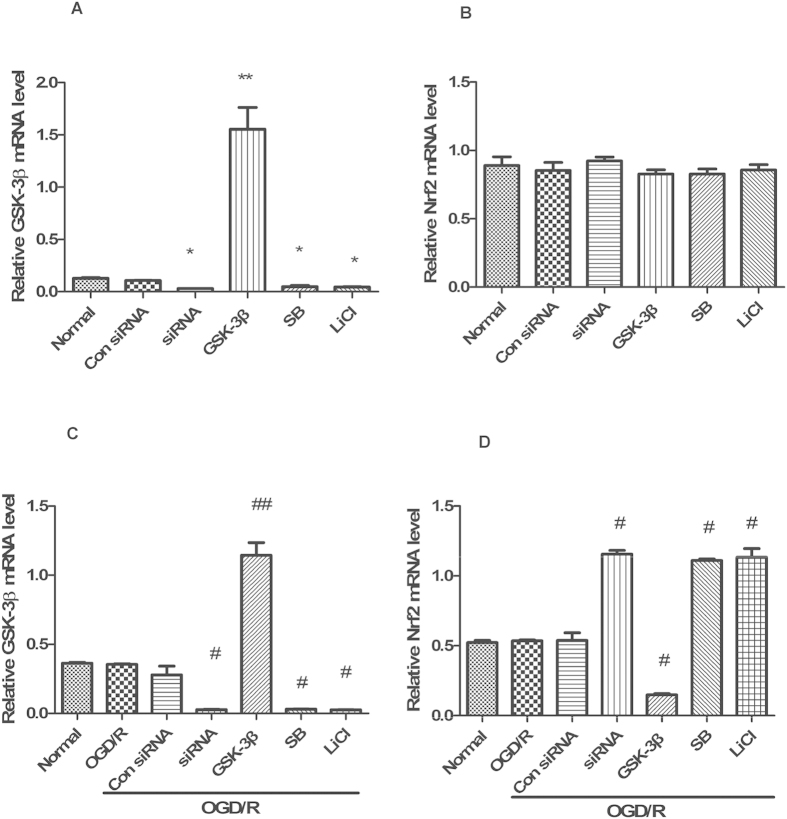
Quantitative RT-PCR analysis of GSK-3β and Nrf2 mRNA levels in neurons. (**A,B**) GSK-3β and Nrf2 mRNA levels analyzed by quantitative RT-PCR from neurons in Fig. 2. (**C,D**) GSK-3β and Nrf2 mRNA levels analyzed by quantitative RT-PCR from neurons in Fig. 3. Bars represent mean ± SEM (n = 4–6). *p < 0.05 vs. Normal. **p < 0.01 vs. Normal, ^#^p < 0.05 vs. OGD/R, ^##^p < 0.01 vs. OGD/R. OGD/R = oxygen and glucose deprivation/reoxygenation.

Prior to isolating total protein, nuclear protein, and RNA from neurons at 6 d, GSK-3β inhibitors (SB 216763 and LiCl) were continuously applied from 6 h, and GSK-3β siRNA and the overexpression lentivirus (GSK-3β) were continuously applied from 72 h. Under normal conditions, treatment with GSK-3β siRNA or GSK-3β inhibitors remarkably decreased expression of both GSK-3β and p- GSK-3β (tyr216) compare with the normal group (Fig. 2A). No statistical difference in total and nuclear Nrf2 expression was observed in the GSK-3β siRNA and inhibitor groups (Fig. 2D) compared with the normal group. This suggests that GSK-3β does not regulate Nrf2 under normal conditions. However, after OGD/R, in the GSK-3β siRNA + OGD/R group and GSK-3β inhibitors + OGD/R groups, expression of total and nuclear Nrf2 significantly increased compared with the OGD/R group (Fig. 3D). The GSK-3β + OGD/R group showed opposite results. The results from real-time fluorescence quantitative (Q-PCR) were consistent with those from western blots (Fig. 4). These results suggest that, after OGD/R, GSK-3β may impose negative regulation on Nrf2 in neurons.

### GSK-3β regulates Nrf2-ARE binding activity in neurons after OGD/R

**Figure 5:**
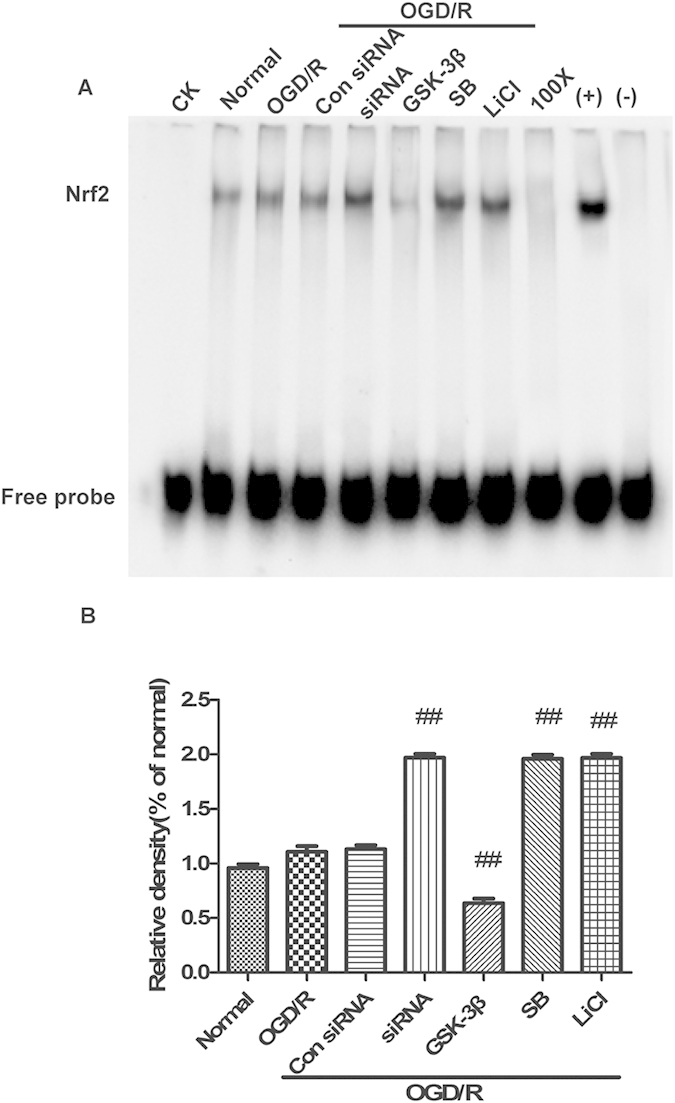
GSK-3β regulates Nrf2-ARE binding in neurons after glucose deprivation/reoxygenation (OGD/R). (**A**) Electrophoretic Mobility Shift Assay (EMSA) analysis of Nrf2-ARE binding. (**B**) Semiquantitative analysis of Nrf2-ARE binding. CK, 100x, (+) and (−) indicate controls. Bars represent mean ± SEM (n = 4–6). ^##^p < 0.01 vs. OGD/R.

Neuronal nuclear extracts were subject to EMSA for measurement of Nrf2-ARE binding activity. There was no statistically significant difference between normal, OGD/R, and control siRNA (con siRNA) + OGD/R groups. Treatment with GSK-3β siRNA + OGD/R and inhibitors + OGD/R resulted in a higher Nrf2 binding activity compared with the OGD/R group (Fig. 5). Opposite results were obtained with the GSK-3β + OGD/R group. These results suggest that after OGD/R, GSK-3β may exert negative control on Nrf2-ARE binding.

### GSK-3β regulates Nrf2/ARE-driven gene expression in neurons after OGD/R

**Figure 6:**
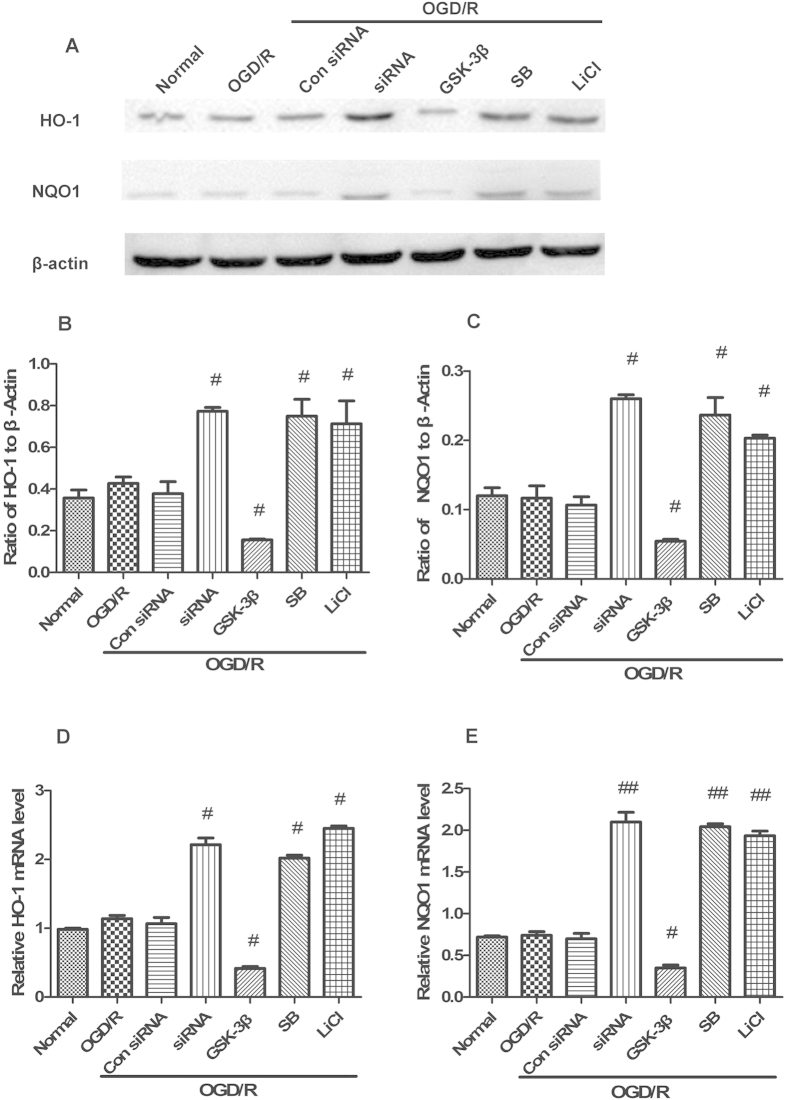
GSK-3β regulates Nrf2/ARE-driven genes in neurons after oxygen and glucose deprivation/reoxygenation (OGD/R). Protein and RNA were collected after OGD for 1.5 h and reoxygenation for 1 h. (**A**) Western blot analysis of HO-1 and NQO1. (**B,C**) Representative ratios of HO-1 and NQO1 to β-actin. (**D,E**) Representative HO-1 and NQO1 mRNA levels analyzed by quantitative RT-PCR. The expression levels of HO-1 and NQO1 significantly increased in the siRNA (GSK-3β siRNA) + OGD/R and inhibitors + OGD/R groups. Reverse results were obtained in the GSK-3β + OGD/R group. Results from quantitative RT-PCR were consistent with those from western blot analysis. Bars represent mean ± SEM (n = 4–6). ^#^p < 0.05 vs. OGD/R, ^##^p < 0.01 vs. OGD/R.

Western blot analysis and Q-PCR were used to investigate the effects of GSK-3β on expression of the Nrf2/ARE-driven genes, HO-1 and NQO1 (Fig. 6). In the GSK-3β siRNA + OGD/R group, expression levels of HO-1 and NQO1 increased by about 1.8-fold and 2.2-fold, respectively, compared with the OGD/R group. In the GSK-3β inhibitors + OGD/R groups, HO-1 and NQO1 expression levels were elevated by about 2-fold and 1.83-fold, respectively, compared with the OGD/R group (Fig. 6A). In the GSK-3β + OGD/R group, HO-1 and NQO1 expression levels decreased by about 2.8-fold and 2.2-fold, respectively. Results from Q-PCR were consistent with those from western blot analysis (Fig. 6D,E). These results suggest that after OGD/R, GSK-3β downregulates expression of Nrf2/ARE-driven genes, including HO-1 and NQO1.

### Expression levels of P-GSK-3β (tyr216), total GSK-3β, β-catenin, and Nrf2 varied with reperfusion time in the cerebral cortex of rats

**Figure 7:**
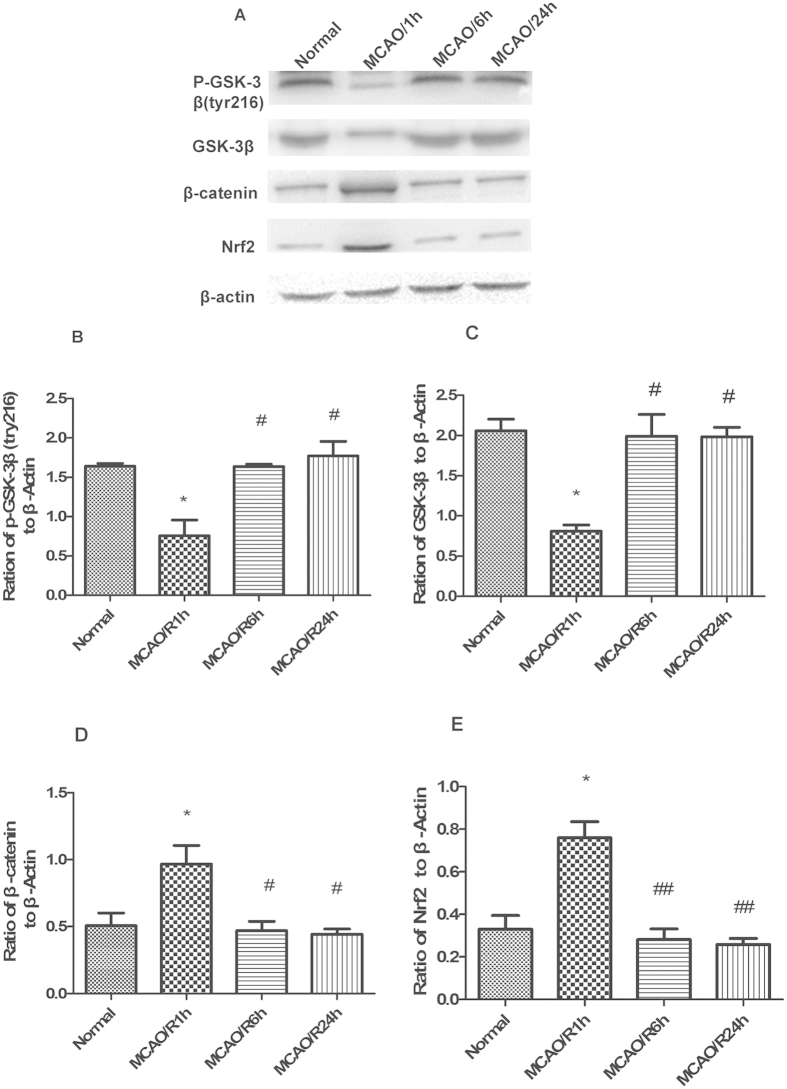
P-GSK-3β (tyr216), total GSK-3β, β-catenin, and Nrf2 expression levels varied with reperfusion time in the cerebral cortex of rats. Rats were subjected to 1 h of middle cerebral artery occlusion (MCAO), followed by 1 h, 6 h, or 24 h of reperfusion. (**A**) Western blot analysis of p-GSK-3β (tyr216), total GSK-3β, β-catenin, and Nrf2. (**B–E**) Representative ratios of p-GSK-3β (tyr216), total GSK-3β, β-catenin, and Nrf2 to β-actin. P-GSK-3β (tyr216) underwent a short-term decrease after 1 h of reperfusion and then initially reached the highest level after 6 h of reperfusion. β-catenin and Nrf2 expression levels showed a reverse trend. Bars represent mean ± SEM (n = 4–6). *p < 0.05 vs. Normal, ^#^p < 0.05 vs. MCAO/R 1 h, ^##^p < 0.01 vs. MCAO/R 1 h.

Rats were subjected to 1 h of MCAO, followed by 1 h, 6 h, or 24 h of reperfusion (Fig. 7). Expression levels of p-GSK-3β (tyr216) were examined using western blot analysis (Fig. 7A,B). Compared with the normal group, the expression level of p-GSK-3β (tyr216) decreased in the 1 h reperfusion group (1.64 ± 0.035 vs. 0.76 ± 0.19, respectively). The expression level of p-GSK-3β (tyr216) significantly increased to 1.64 ± 0.05 in the 6 h reperfusion group, and remained at this level in the 24 h group (1.77 ± 0.078). Total GSK-3β expression showed a similar trend (Fig. 7A). Expression of β-catenin yielded opposite results (Fig. 7A). We also found that Nrf2 expression showed a reverse trend (Fig. 7A). After 1 h of reperfusion, the level of Nrf2 expression increased about 2.3-fold compared with the normal group (0.76 ± 0.075 vs. 0.33 ± 0.06). After 6 h and 24 h of reperfusion, expression of Nrf2 (0.28 ± 0.04, 0.26 ± 0.02, respectively) decreased to the normal level (0.33 ± 0.06). The expression of P-GSK-3β (tyr216) initially reached the highest level after 6 h of reperfusion. Therefore, to analyze GSK-3β regulation of Nrf2 expression, 6 h of reperfusion was used for subsequent experiments.

### GSK-3β regulates Nrf2 in the cerebral cortex of rats after MCAO/R

**Figure 8:**
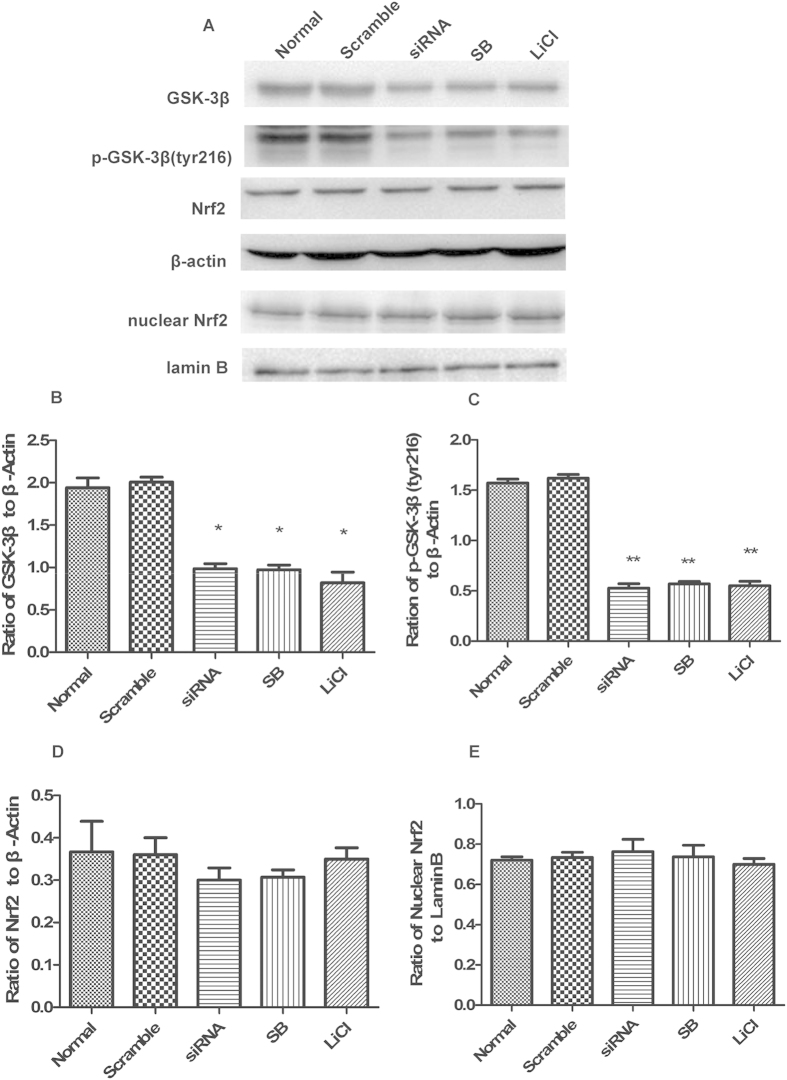
GSK-3β regulation of Nrf2 in the cerebral cortex of rats in normal conditions. (**A**) Western blot analyses of GSK-3β, p-GSK-3β (tyr216), Nrf2, and nuclear Nrf2. (**B–E**) Representative ratios of GSK-3β, p-GSK-3β (tyr216), Nrf2, and nuclear Nrf2 to β-actin. GSK-3β and p-GSK-3β (tyr216) expression significantly decreased in the siRNA (GSK-3β siRNA) and inhibitor [SB(SB216763) and LiCl] groups compared with the normal group. There was no significant difference in the expression levels of Nrf2 and nuclear Nrf2 in the siRNA and inhibitor groups compared with the normal group. Bars represent mean ± SEM (n = 4–6). *p < 0.05 vs. Normal, **p < 0.01 vs. Normal.

**Figure 9:**
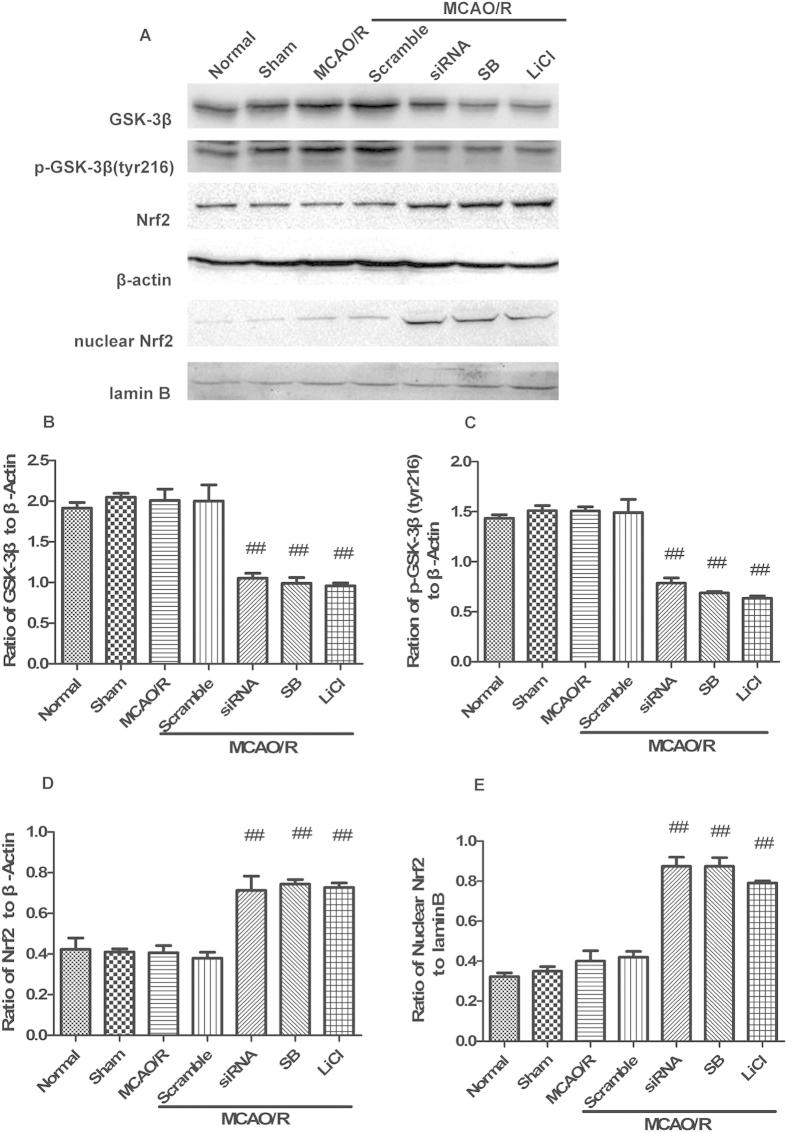
GSK-3β regulates Nrf2 in the cerebral cortex of rats after middle cerebral artery occlusion-reperfusion (MCAO/R). Rats were subjected to MCAO for 1 h followed by 6 h of reperfusion. (**A**) Western blot analysis of GSK-3β, p-GSK-3β (tyr216), Nrf2, and nuclear Nrf2. (**B–E**) Representative ratios of GSK-3β, p-GSK-3β (tyr216), Nrf2, and nuclear Nrf2 to β-actin. GSK-3β and p-GSK-3β (tyr216) expression significantly decreased in the siRNA + MCAO/R and inhibitors + MCAO/R groups compared with the MCAO/R group. Expression levels of Nrf2 and nuclear Nrf2 significantly increased in the siRNA + MCAO/R and inhibitors + MCAO/R groups. Bars represent mean ± SEM (n = 4–6). ^##^P < 0.01 vs. MCAO/R.

**Figure 10:**
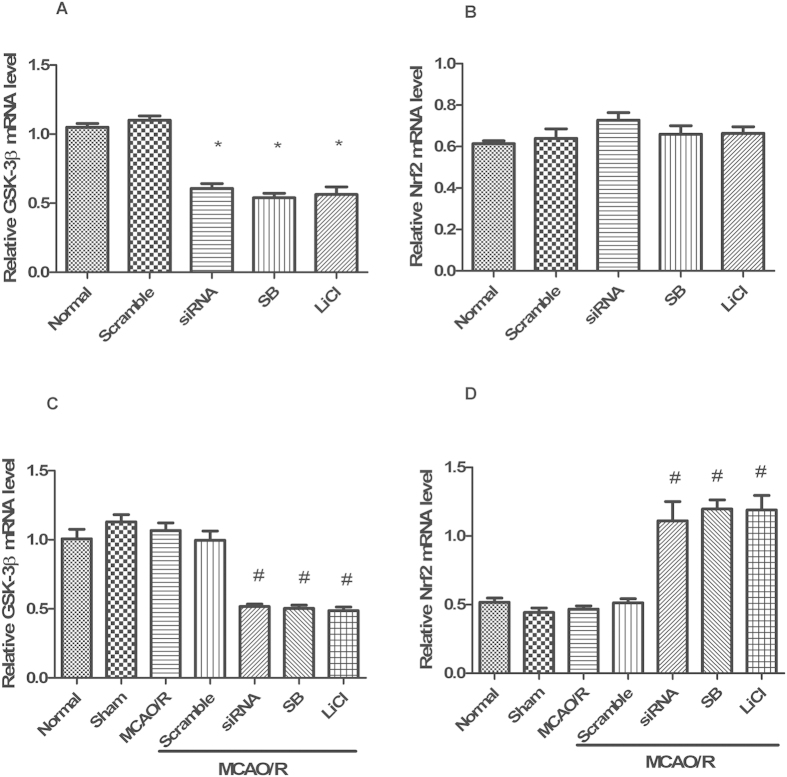
Quantitative RT-PCR analysis of GSK-3β and Nrf2 mRNA levels in the cerebral cortex of rats. (**A**,**B**) GSK-3β and Nrf2 mRNA levels analyzed by quantitative RT-PCR from the cerebral cortex in Fig. 8. (**C,D**) GSK-3β and Nrf2 mRNA levels analyzed by quantitative RT-PCR from the cerebral cortex in Fig. 9. Bars represent mean ± SEM (n = 4–6). *p < 0.05 vs. Normal, ^#^p < 0.05 vs. MCAO/R. MCAO/R = middle cerebral artery occlusion-reperfusion.

Under normal conditions, treatment with GSK-3β siRNA or GSK-3β inhibitors significantly decreased both GSK-3β and p-GSK-3β (tyr216) levels compared with the normal group (Fig. 8A). Total and nuclear Nrf2 expression showed almost no significant change (Fig. 8A). Thus, GSK-3β does not regulate Nrf2 under normal conditions. However, after 1 h of MCAO followed by 6 h of reperfusion, total Nrf2 expression significantly increased approximately 1.8-fold in the GSK-3β siRNA + MCAO/R and GSK-3β inhibitors + MCAO/R groups, compared with the MCAO/R group (Fig. 9A). In addition, nuclear Nrf2 expression significantly increased approximately 2-fold (Fig. 9A). The results from Q-PCR were consistent with those from western blot analysis (Fig. 10). These results suggest that GSK-3β does not regulate Nrf2 under normal conditions. However, after MCAO/R, GSK-3β downregulates Nrf2 in the cerebral cortex of rats. These results are consistent with those from our *in vitro* experiments.

### GSK-3β regulates Nrf2-ARE binding activity in the cerebral cortex of rats after MCAO/R

**Figure 11:**
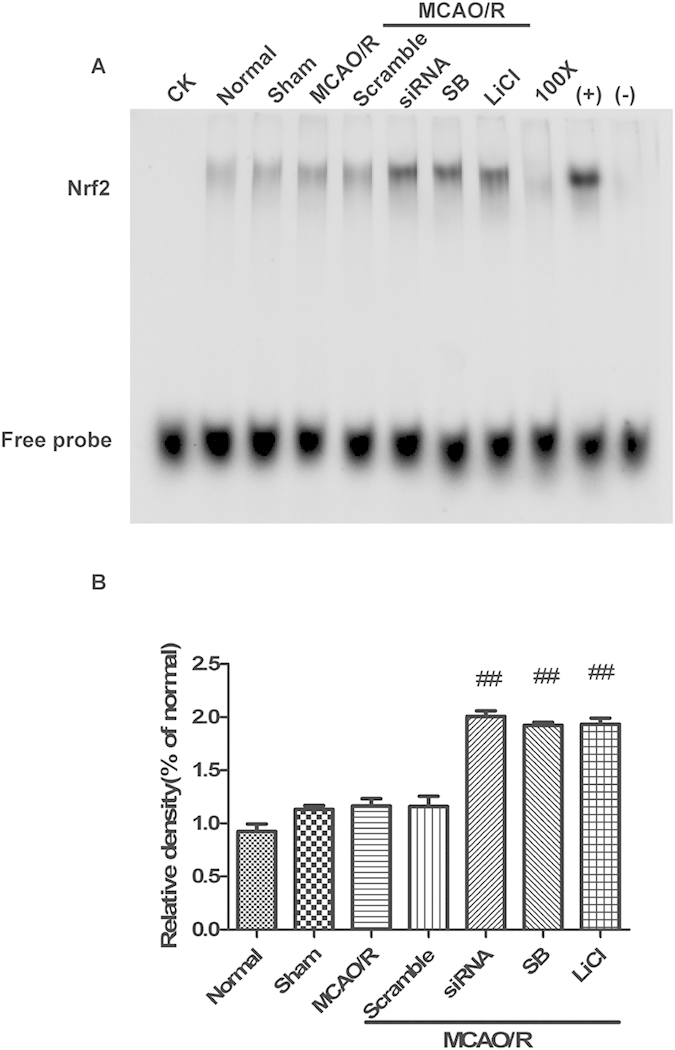
GSK-3β regulates Nrf2-ARE binding activity in the cerebral cortex of rats after middle cerebral artery occlusion-reperfusion (MCAO/R). (**A**) Electrophoretic Mobility Shift Assay (EMSA) analysis of Nrf2-ARE binding. (**B**) Semiquantitative analysis of Nrf2-ARE binding. CK, 100x, (+) and (−) indicate different controls. Bars represent mean ± SEM (n = 4–6). ^##^p < 0.01 vs. MCAO/R.

Nuclear extracts from the cerebral cortex were subjected to EMSA for measurement of Nrf2-ARE binding. Inhibiting GSK-3β by transfecting with GSK-3β siRNA and treating with inhibitors significantly increased Nrf2-ARE binding activity after MCAO/R (Fig. 11). These results suggest that GSK-3β negativity regulates Nrf2-ARE binding in the cerebral cortex of rats after MCAO/R. This result is consistent with our *in vitro* experiments.

### GSK-3β regulates expression of Nrf2/ARE-driven genes in the cerebral cortex of rats after MCAO/R

**Figure 12:**
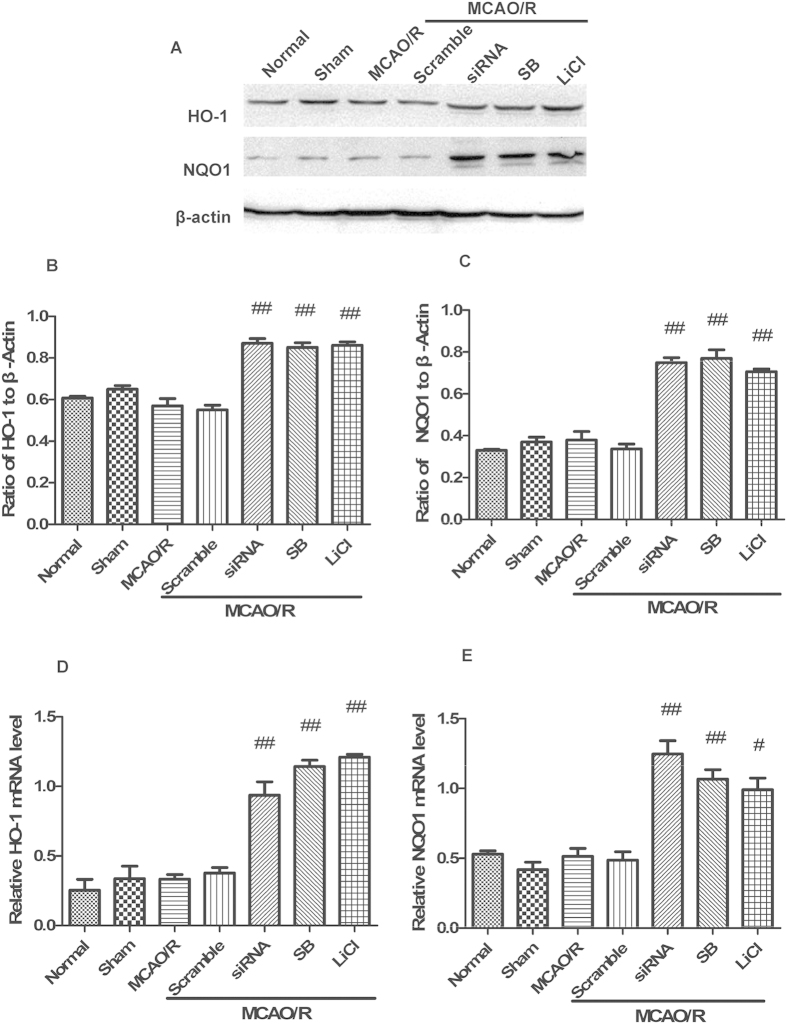
GSK-3β regulates Nrf2/ARE-driven genes in the cerebral cortex of rats after middle cerebral artery occlusion-reperfusion (MCAO/R). Protein and RNA were collected after MCAO for 1 h and reperfusion for 6 h. (**A**) Western blot analysis of HO-1 and NQO1. (**B,C**) Representative ratios of HO-1 and NQO1 to β-actin. (**D,E**) Representative HO-1 and NQO1 mRNA levels analyzed by quantitative RT-PCR. Expression levels of HO-1 and NQO1 significantly increased in the siRNA and inhibitor groups. Results from quantitative RT-PCR were consistent with those from western blot analysis. Bars represent mean ± SEM (n = 4–6). ^#^p < 0.05 vs. MCAO/R, ^##^P < 0.01 vs. MCAO/R.

After 6 h of reperfusion, expression levels of the Nrf2/ARE-driven genes, HO-1 and NQO1, were analyzed by western blot and Q-PCR (Fig. 12). In the GSK-3β siRNA + MCAO/R group, expression levels of HO-1 and NQO1 significantly increased approximately 1.5-fold and 2-fold, respectively, compared with the MCAO/R group (Fig. 12A). In the GSK-3β inhibitors + MCAO/R groups, HO-1 expression levels significantly increased about 1.5-fold, and NQO1 expression levels significantly increased about 1.9-fold (Fig. 12A). The results from Q-PCR were consistent with those from western blot analysis (Fig. 12D,E). These results suggest that GSK-3β downregulates expression of Nrf2/ARE-driven genes, including HO-1 and NQO1 in the cerebral cortex of rats after MCAO/R. These results are consistent with our *in vitro* experiments.

## Discussion

In the present study, we explored the relationship between GSK-3β and Nrf2 in neurons that were subjected to OGD/R and in the cerebral cortex of rats that sustained MCAO/R. We showed that the activity of GSK-3β in neurons underwent a short-term decrease at 0.5 h of reoxygenation and then increased at 1 h of reoxygenation. Similarly, the activity of GSK-3β in the cerebral cortex of rats decreased at 1 h of reperfusion and then increased at 6 h of reperfusion. Nrf2 expression showed an opposite trend *in vitro* and *in vivo*. We employed siRNA knockdown and inhibition of GSK-3β *in vivo* and *in vitro*, which increased expression of Nrf2, Nrf2-ARE binding, and expression of antioxidant proteins HO-1 and NQO1 that are downstream from ARE. Overexpression of GSK-3β in cerebral neurons of rats demonstrated a reverse tendency. Our results suggest that increasing the level of activated GSK-3β inhibits the Nrf2/ARE signaling pathway of ischemia-reperfusion in the cerebral cortex and OGD/R in neurons. Thus, GSK-3β is a negative regulatory factor for Nrf2 in cerebral ischemia-reperfusion and OGD/R in neurons.

Nrf2, a key transcription factor, is involved in expression of many cytoprotective genes. It is well known that Nrf2 is normally retained in the cytoplasm by Keap1^13^,^14^,^15^. In the present study, we focused on GSK-3β, which is a multifunctional kinase. GSK-3β has drawn considerable attention in recent years. It is a key kinase that is involved in several cellular signaling pathways and sensitizes cells for cell death. Recent research indicates that GSK-3β, as a negative regulator of Nrf2, participates in the distribution of Nrf2 inside and outside of the nucleus^16^,^17^. GSK-3β regulation of Nrf2 transcription activity is independent of the expression of Keap1^9^,^18^. In addition, inhibition of GSK-3β before ischemia or just before reperfusion has been shown to reduce myocardial infarct size^19^,^20^,^21^,^35^. Moreover, research has also demonstrated that inhibition of GSK-3β improves cognition during oxidative stress in a mouse model of Alzheimer’s disease. This effect may coincide with reduced nuclear translocation of Nrf2^22^,^23^,^24^. In this study, we demonstrated that inhibiting GSK-3β could induce accumulation of Nrf2 in the nucleus. However, the effects of GSK-3β on Nrf2 and the Nrf2/ARE signaling pathway in cerebral ischemia-reperfusion had not been studied previously. Nrf2 translocates to the nucleus where it activates the ARE of phase II detoxifying enzymes and antioxidant stress genes such as NQO1, and accelerates their transcription and expression. Nrf2 accumulation in the nucleus can guard against oxidative stress. However, Nrf2 cannot always be located in the nucleus; Nrf2 will be exported out of the nucleus and degraded. Studies have shown that GSK-3β leads to Nrf2 nuclear export and degradation through 2 pathways. Suryakant (2011) reported that activated GSK-3β controls accumulation of Src kinases in the nucleus. Nuclear accumulation of Src kinases results in phosphorylation of Nrf2 (Tyr568), which leads to Nrf2 nuclear export and degradation in mouse hepatoma (Hepa-1) cells^25^. Partricia (2011) reported that activation of GSK-3 based on phosphorylation of the Neh6 domain of Nrf2 and ubiquitination degraded Nrf2 through the β-TrCP/Cullin1 E3 ligase complex in human embryonic kidney (HEK) 293 T cells^9^.

OGD/R was used to mimic ischemic reperfusion insult *in vitro*. GSK-3β was activated by phosphorylation of Tyr-216 [p-GSK-3β (tyr216)]. The activity of GSK-3β was detected indirectly by examining β-catenin. We observed that activated GSK-3β decreased after 0.5 h of reperfusion, yet increased after 1 h of reperfusion. It is possible that the PI3K/Akt pathway is activated during the early phase of antioxidant stress, involving short-term activation of Akt and inhibition of GSK-3β by phosphorylation of GSK-3β (Ser 9)^26^. After long-term antioxidant stress, inhibition of GSK-3β by the PI3K/Akt pathway decreases, possibly due to an unknown tyrosine kinase that activates GSK-3β, as well as increased expression of GSK-3β^25^. Expression of Nrf2 changed in the opposite direction. However, Jaiswal (2006) reported that H_2_O_2_ activated GSK-3β after 4 h in HepG2 cells^13^. It is possible that the differences in time course were due to differences in sensitivity to oxidative stress between cerebral neurons and HepG2 cells. GSK-3β interference or inhibition increased total Nrf2 protein, accumulation in the nucleus, as well as Nrf2 mRNA. These results agree with a study by Abhinav (2007), which showed that continuous activation of GSK-3β prevented accumulation of Nrf2 in the nucleus when HepG2 cells were transfected with GSK-3β siRNA^16^. Knockdown or inhibition of GSK-3β increased Nrf2-ARE binding as shown by EMSA. Furthermore, knockdown or inhibition of GSK-3β activated expression of genes downstream from ARE, including HO-1 and NQO1. Overexpression of GSK-3β yielded opposite results in neurons. These results are similar to those of Salazar *et al*. (2006), who demonstrated that transcription of phase II detoxifying enzymes and antioxidants significantly decreased when cotransfected with Nrf2 and GSK-3β in HEK 293 T cells^27^,^36^. In addition, we observed that GSK-3β was activated after 6 h of reperfusion *in vivo*. The effects of interfering or inhibiting GSK-3β before MCAO in rats showed changes similar to the study *in vitro*. The results described above were not observed under normal conditions both *in vivo* and *in vitro*. It is possible that under normal conditions, GSK-3β is inhibited by phosphorylation at GSK-3β Ser-9, and regulation and degradation of Nrf2 occurs primarily through Keap1/Cullin 3/Rbx1 complexes^9^.

In this study, we demonstrated negative regulation of the transcription factor Nrf2 by GSK-3β after oxidative stress induced by OGD/R *in vitro* and cerebral ischemia-reperfusion *in vivo*. Our research suggests a potential new direction for treatment of stroke. However, the specific mechanisms through which GSK-3β regulates Nrf2 have not been clarified in cerebral ischemia-reperfusion. Whether or not GSK-3β regulates Nrf2 through the Src subfamily of kinases and the β-TrCP pathway requires further study.

## Methods and Materials

### Experimental Animals and Chemicals

Adult male Sprague-Dawley rats (60–80 d old, 240–300 g) were used for the *in vivo* study. Newborn Sprague-Dawley rats (0–24 h old) were used to culture primary cortical neurons. The animal protocol was approved by the Chongqing Medical University Biomedical Ethics Committee. All experimental procedures were performed in accordance with the National Institutes of Health Guide for the Care and Use of Laboratory Animals. All efforts were made to minimize the number of animals used and their suffering.

SB216763 and LiCl were purchased from Sigma-Aldrich (St Louis, MO, USA). SB216763 was dissolved in dimethyl sulfoxide (DMSO) and diluted with saline. LiCl was dissolved in saline.

### Primary Culture of Rat Cortical Neurons and OGD/R

Neurons were cultured as described in our previous studies^28^,^29^. Cortical neurons were obtained from the cerebral cortex of 24-h-old rats. Approximately 2 × 10^6^ cells in 2 mL of Neurobasal Medium containing glutamine (1 mM), 1% penicillin and streptomycin (Pen/Strep) (penicillin 100 U/mL, streptomycin 100 μg/mL), and 2% B27 supplement were seeded per well. Neurons were cultured in a humidified incubator with 5% CO_2_/balanced with air (result: 20% O_2_) at 37 °C. The cells were cultured for 6–7 d *in vitro*. Cultured cells were examined using NeuN and GFAP staining to ensure that more than 90% of the cells were neurons. OGD/R was conducted as previously described^30^,^31^. Briefly, after neurons were cultured for 6 d, they were washed 3 times with glucose-free DMEM. The glucose-free DMEM had been previously equilibrated with 1% O_2_, 5% CO_2_, and 94% N_2_ at 37 °C in an incubator. Neurobasal Medium was then replaced with glucose-free DMEM, and the cells were transferred to an incubator with 1% O_2_, 5% CO_2_, and 94% N_2_ for 1.5 h at 37 °C. The medium was then changed back to Neurobasal Medium and the cultures were returned to the normal incubator for recovery times of 0.5 h, 1 h, 4 h, or 6 h. An appropriate time of reoxygenation was selected for subsequent studies.

Cultured neurons were divided into 12 groups. Under normal culture conditions, the groups were: normal group, control siRNA (con siRNA) group, GSK-3β siRNA (siRNA) group, GSK-3β overexpression (GSK-3β) group, SB216763 (SB) group, and the LiCl group. Following OGD/R, the groups were: OGD/R group, con siRNA + OGD/R group, GSK-3β siRNA + OGD/R group, GSK-3β + OGD/R group, SB + OGD/R group, and the LiCl + OGD/R group.

### GSK-3β Overexpression and Interference in Neurons

Neuron Biotech (Shanghai, China) constructed pcDNA-GSK-3β (NCBI accession no. NM 032080.1). The GSK-3β overexpression lentivirus was packaged with pLOV-UbiC-EGFP plasmid and transfected into 293 T cells. The GSK-3β viral supernatant (Lenti-GSK-3β) was harvested, filtered, and concentrated^32^.

Synthetic oligonucleotides containing the rat GSK-3β splice variant RNA interference target GCTAGATCACTGTAACATAGT were packaged with the pLKD.UbiC.GFP lentiviral vector (Neuron Biotech). The oligonucleotide sequences were: 5ʹ-CCGGGCTAGATCACTGTAA-CATAGTCTCGAGACTAT

GTTACAGTGATCTAGCTTTTTTG-3ʹ(sense); 5ʹ-AATTCAAAAAAGCTAGATCACTGTAACATAGTCTCG-AGACTATGTTACAGTGATCTAGC-3ʹ(antisense). The negative control sequence 5′-TCAGACTTGATACTGAACTGA-3′was also supplied by Neuron Biotech.

After neurons were cultured for 3 days, cells were infected with Lenti-GSK-3β or Lenti-GSK-3β-RNAi at a multiplicity of infection (MOI) of 20 in medium. GFP-positive cells were confirmed under a fluorescence microscope. Sustained GSK-3β overexpression or downregulation were confirmed by qRT-PCR and western blot analysis 72 h after transfection.

### MCAO and Design of *In Vivo* Experiments

Rats were given free access to food and water in optimal surroundings before the operation. Adult rats were divided randomly into 11 groups. Under normal conditions, the groups were: normal group, scramble group, GSK-3β siRNA (siRNA) group, SB216763 (SB) group (20 μg/kg, intracerebroventricular injection), and the LiCl group (50 mg/kg, intraperitoneal injection). After MCAO/R, the groups were: sham-operated (sham) group, MCAO/R group, scramble + MCAO/R group, GSK-3β siRNA (siRNA) + MCAO/R group, SB + MCAO/R group, and the LiCl MCAO/R group. Transient cerebral ischemia (MCAO) was described in detail in our previous study^33^,^34^. Rats were anesthetized with chloral hydrate (350 mg/kg, intraperitoneal injection) and subjected to the operation. A nylon filament (diameter 0.24–0.28 mm) was inserted into the middle cerebral artery for 1 h. The nylon filament was carefully removed to allow blood to return to the ischemic artery, and then was sutured to establish reperfusion. Regional cerebral blood flow was detected by an ultrasonic blood flow meter before ischemia, during MCAO, and during reperfusion. Sham-operated rats were subjected to the same surgical procedure as MCAO rats except for occlusion of the common carotid arteries. Animals that had blood reperfusion below 70% or that died during reperfusion were excluded from analysis.

### GSK-3β Interference in Rats

GSK-3β siRNA was constructed by Shanghai GenePharma Co., Ltd (forward 5ʹ-GGAGAGCCCAAUGUUUCAUTT-3ʹand reverse 5ʹ-AUGAAACAUUGGGCUCUCCTT-3ʹ). SiRNAs were dissolved in RNase-free water to a final concentration of 2 μg/μL. Forty-eight hours before MCAO, 7 μL of GSK-3β siRNA was injected ipsilaterally into the left lateral cerebral ventricle. Transfection efficiency was confirmed under a fluorescence microscope. As a control, rats were injected with the scramble siRNA (forward 5ʹ-GCGCCAGUGGUACUUAAUATT-3ʹand reverse 5ʹ-UAUUAAGUACCACUGGCGCTT-3ʹ) using the same procedure as for GSK-3β siRNA. Sustained GSK-3β downregulation was confirmed by qRT-PCR and western blot analysis 48 h after transfection.

### Western Immunoblot Analysis

Total protein was extracted from cultured neurons and the ischemic penumbra of the rat cortex using cell lysis buffer supplemented with proteinase and phosphatase inhibitors. The nuclear proteins were extracted using a commercial kit (Beyotime, China). Cell lysates were separated by 10% SDS-PAGE and transferred to polyvinylidene fluoride (PVDF) membranes. The membranes were then blocked in 5% non-fat milk TBST buffer for 1.5 h at room temperature. The membranes were incubated in primary antibody overnight at 4 °C and in secondary antibody for 1 h at room temperature. Dilutions for primary antibodies were as follows: anti-GSK-3β (#9315, 1:1000, Cell Signaling Technology, Boston, MA, USA), anti-β-catenin (#9582, 1:1000, Cell Signaling Technology), anti-Nrf2 (YT3189, 1:500, Immunoway, Houston, TX, USA), anti-GSK-3β (phospho-tyr216) (ab75745, 1:500, Abcam, Cambridge, MA, USA), anti-LaminB1 (ab133741, 1:500, Abcam), anti-HO-1 (BS6626, 1:500, Bioworld, St. Louis Park, Minnesota, USA), anti-NQO1 (BS6833, 1:500, Bioworld), and anti-actin (BS 6007 M, 1:10,000, Bioworld).The secondary antibody was diluted 1:5000 (Sangon Biotech, S hanghai, Co., Ltd.). The density of bands was detected using an imaging densitometer (Bio-Rad, Foster City, CA, USA), and the gray value of bands was quantified using Quantity One 1-D analysis software.

### qRT-PCR

Total RNA was extracted using RNAiso Plus (TaKaRa Biotechnology, Dalian, China) according to the manufacturer’s protocol. Next, reverse transcription was performed using a cDNA synthesis kit (TaKaRa Biotechnology). Real-Time PCR reactions were conducted using TaKaRa SYBR Premix Ex Taq II (Tli RnaseH Plus) (TaKaRa Biotechnology) on a PCR amplifier (CFX-96 Content Real-time System). Primers are listed in the Table 1. (Sangon Biotech, Shanghai, Co., Ltd.).

### Electrophoretic Mobility Shift Assay (EMSA)

EMSA was conducted using BiotinLight^TM^ EMSA Kit (Exprogen, China). For EMSA, The Nrf2 consensus oligonucleotide probe (Bio-5ʹ-TGG GGA ACC TGT GCT GAG TCA CTG GAG-3ʹ) was end-labeled with [c-32 P] ATP (Sangon Biotech) using T4-polynucleotide kinase. Five micrograms of neuronal total nuclear protein or 8 μg of cerebral nuclear proteins were incubated in a binding buffer for 20 min at room temperature. Samples were then loaded on a non-denaturing 6.5% polyacrylamide gel and electrophoretically separated in 0.25X TBE buffer. The gel was vacuum-dried and exposed to X-ray film (Fuji Hyperfilm, Tokyo, Japan) at 80 °C with an intensifying screen. Levels of Nrf2 DNA binding activity were quantified by computer-assisted densitometric analysis.

### Statistical Analysis

All data are expressed as mean ± S.E.M. One-way analysis of variance (ANOVA) followed by Student’s *t* test was used to compare results among all groups. The SPSS 11.5 software^35^,^36^ package was used to perform all statistics. P < 0.05 was considered statistically significant.

## Additional Information

**How to cite this article**: Chen, X. *et al*. GSK-3β downregulates Nrf2 in cultured cortical neurons and in a rat model of cerebral ischemia-reperfusion. *Sci. Rep*. **6**, 20196; doi: 10.1038/srep20196 (2016).

## Figures and Tables

**Table 1:** Primers used in qRT-OCR.

Gene product	Forward primer	Reverse primer	Fragment size (*bp*)
Nrf2	5′-ATCCAGACAGACACCAGTGGATC-3′	5′-GGCAGTGAAGACTGAACTTTCA-3′	179
GSK-3β	5′-TACCCATACGATGTTCCAGAT-3′	5′-ACCCTGCCCAGGAGTTGCCAC-3′	120
HO-1	5′-TGCTCAACATCCAGCTCTTTGA-3′	5′-GCAGAATCTTGCACTTTGTTGCT-3′	120
NQO1	5′-AGGCTGGTTTGAGCGAGT-3′	5′-ATTGAATTCGGGCGTCTGCTG-3′	269
β-actin	5′-TGTTTGAGACCTTCAACACC-3′	5′-CGCTCATTGCCGATAGTGAT-3′	207
